# *NLRP1* and *NLRP3* polymorphisms in mesothelioma patients and asbestos exposed individuals a population-based autopsy study from North East Italy

**DOI:** 10.1186/s13027-015-0022-0

**Published:** 2015-08-01

**Authors:** Violetta Borelli, Ronal R Moura, Elisa Trevisan, Sergio Crovella

**Affiliations:** Department of Life Sciences, University of Trieste, Trieste, Italy; Department of Genetics, Federal University of Pernambuco, Recife, Brazil; Department of Medical, Surgical and Health Sciences, University of Trieste, Trieste, Italy

**Keywords:** Mesothelioma, Inflammasome, *NLRP1* and *NLRP3* polymorphisms, Asbestos exposure, Haplotypes

## Abstract

*NRLP1* (rs12150220, rs9889625, rs9900356, rs6502867, rs2670660) and *NLRP3* (rs35829419, rs10754558) polymorphisms have been analyzed in 69 subjects with documented asbestos exposure and death for malignant pleural mesothelioma and 59 patients with documented asbestos exposure but death for other causes, all from a North East Italy. No association was found between *NLRP1* and *NLRP3* polymorphisms and susceptibility to develop mesothelioma using the general, dominant or recessive models. Also haplotype analysis did not reveal any significant association with mesothelioma. Our findings, being controversial with respect to another study on Italian patients, do suggest the need of further studies to unravel the contribution of *NLRP1* and *NLRP3* in susceptibility to mesothelioma.

**Electronic supplementary material:**

The online version of this article (doi:10.1186/s13027-015-0022-0) contains supplementary material, which is available to authorized users.

## Findings

The involvement of inflammasome in susceptibility to develop malignant pleural mesothelioma (MPM), considering its capacity to sense asbestos fibres has been already reported [1] and several papers have been published so far on this topic [2–4].

Genetic susceptibility to mesothelioma has been widely analyzed and some recent genome wide studies pointed out several susceptibility genes (i.e. *BAP1, NF2, CDKN2A, CUL1, SLC7A14, THRB, CEBP350, ADAMTS2, ETV1, PVT1* and *MMP14*), but genetic variants in inflammasome genes such as *NLRP3* and *NLRP1* have not been associated to mesothelioma [5–7]. However, Girardelli et al. [8] reported an association of rs12150220 *NLRP1* polymorphism with mesothelioma in Italian patients the presence of T allele conferred a major risk (OR 1.79) to acquire the disease.

With the aim of replicating Girardelli et al. [8] results, we analyzed *NRLP1* and *NLRP3* polymorphisms in subjects exposed to asbestos from North East Italy.

We collected 128 subjects deceased during the period 1980–2000, of which 69 with documented asbestos exposure and death for MPM (AEM) and 59 patients with documented asbestos exposure but death for causes different from MPM and other asbestos related diseases (AENM), used as controls. All subjects were characterized based on information available in medical records and the MPM diagnosis was performed by histologic examination. For the AENM group objective signs confirming asbestos exposure were the presence of hyaline plaque at autopsy (≥2°) at pleural level, presence of asbestos bodies (AB) in routine lung sections and (for most cases) counts of AB, expressed as number of AB per gram of dry lung tissue, ≥10.000/g.

Genomic DNA of AEM and AENM was extracted from formalin-fixed and paraffin-embedded archived autopsy heart tissue slices (about 40–50 slices with a thickness of 5–7 μm were cut from the same paraffin block) using the QIAsymphony DSP DNA mini kit (QIAGEN, Hilden, Germany) and eluted in 50ul of TE buffer.

Quantitative and qualitative evaluation of extracted DNA was performed using NanoDrop ND-1000 (Termo Scientific, Wilmington, USA); electrophoresis on agarose gel allowed us to verify the degree of DNA degradation.

We genotyped two *NLRP3* polymorphisms (rs35829419, cSNP and rs10754558, Tag SNP) and five *NLRP1* polymorphisms (rs12150220, cSNP; rs9889625, Tag SNP; rs9900356, Tag SNP; rs6502867, Tag SNP; rs2670660, Tag SNP) using the following Taqman genotyping assays: *NLRP3* C__25648615_10, C__26052028_10; *NLRP1* C___1600653_10, C___1274455_10, C__29796119_10, C__29222211_20 and C___1600689_10. Allelic specific PCR reactions have been run on ABI 7500 real time instrument (Life Technologies, Foster City, CA). SDS software (Life Technologies) allowed allelic discrimination and genotyping.

Table 1 summarizes the distribution of *NLRP1* and *NLRP3* polymorphisms genotype counts among the AEM and AENM groups (a graphic representation is also provided as Additional file 1: Figure S1). All markers were in Hardy-Weinberg Equilibrium (HWE) in both AEM and AENM groups with the exception of *NLRP1* rs9900356 SNP in AEM group and *NLRP3* rs35829419, which was not in HWE in AENM and AEM.

**Table 1:** *NLRP1* and *NLRP3* polymorphisms genotype counts among the AEM and AENM groups

*NLRP1*					
SNP	AEM (n = 69)	AENM (n = 59)	OR	95 % CI	p-value
rs9900356					
T/T	40	34	1.00	Ref	
T/C	25	23	0.92	0.45 - 1.91	0.9781
C/C	4	2	1.70	0.29 - 9.86	0.8645
rs6502867					
T/T	36	34	1.00	Ref	
T/C	23	19	1.14	0.53 - 2.46	0.8835
C/C	10	6	1.57	0.52 - 4.80	0.6008
rs9889625					
G/G	29	25	1.00	Ref	
A/G	25	21	1.03	0.47 - 2.26	0.8911
A/A	15	13	0.99	0.40 - 2.48	0.8242
rs12150220					
A/A	20	18	1.00	Ref	
T/A	29	25	1.04	0.45 - 2.40	0.9118
T/T	20	16	1.13	0.45 - 2.81	0.9849
rs2670660					
A/A	17	15	1.00	Ref	
A/G	34	30	1.00	0.43 - 2.34	0.8283
G/G	18	14	1.13	0.42 - 3.04	1.0000
*NLRP3*					
SNP	AEM (n = 69)	AENM (n = 59)	OR	95 % CI	
rs35829419					
C/C	60	52	1.00	Ref	
A/C	6	6	0.87	0.26 - 2.85	0.9452
A/A	3	1	2.60	0.26 - 25.76	0.7379
rs10754558					
C/C	23	23	1.00	Ref	
C/G	33	25	1.32	0.61 - 2.87	0.6152
G/G	13	11	1.18	0.44 - 3.18	0.9369

*OR* Odds Ratio, *CI* Confidence Interval, *Ref* reference genotype (the most frequent)

No association was found between *NLRP1* and *NLRP3* polymorphisms and susceptibility to develop mesothelioma using the general, dominant or recessive models. Moreover, we evaluated the formation of haplotypes in *NLRP1* and *NLRP3* genes. We detected two blocks for *NLRP1*: Haplo1 (rs9900356, rs6502867 and rs9889625; D’ = 1) and Haplo2 (rs12150220 and rs2670660; D’ = 0.83). The two *NLRP3* SNPs also formed a haplotype (D’ = 1). We estimated the haplotype frequencies of the *NLRP1* and *NLRP3* polymorphisms in AEM and AENM individuals and no association with susceptibility to the disease was found; haplotype distributions are in Additional file 2: Table S1; Additional file 3: Figure S2, reports the haploblocks found for *NLRP1* and *NRLP3* SNPs.

So the association (for both rs12150220 SNP and rs12150220- rs2670660 haplotype) with susceptibility to mesothelioma reported by Girardelli et al. [8] has not been replicated in our study, also performed in Italian patients and controls (our samples were from Monfalcone, an industrial area with endemic exposition to asbestos).

However, in the study of Girardelli et al. *NLRP1* and *NRLP3* polymorphisms have been evaluated in patients with mesothelioma and controls living in a geographic area known to be at risk of asbestos exposure, while in our study both mesothelioma patients and controls had documented asbestos exposure (see Additional file 4: Table S2 for details), so our genetic analysis has been performed on more homogeneous cohorts.

Girardelli et al. [8] et al. hypothesized a role for NLRP1 inflammasome in the context of mesothelioma on the basis of NLRP1 lung expression (alveolar macrophages and epithelial cells), a location where this innate immune protein could act as sensor of asbestos fibres. The authors suggested a possible functional role for *NLRP1* functional SNP rs12150220, indicating the presence of the T allele as able to imbalance NLRP1 production. Since no functional data are already available in the literature for rs12150220 variation, we performed an *in silico* search with SIFT and Polyphen softwares able to predict an eventual detrimental effect of the SNP: both SIFT (0.16) and Polyphen (0.415) predictive values indicate no damaging effect for *NLRP1* rs12150220 SNP. Being aware of the limitations of *in silico* SNPs functional prediction, the hypothesized role of this variation should be functionally verified.

In conclusion, our study, performed on patients and controls with homogeneous asbestos exposure, does not confirm the involvement of NLRP1 inflammasome in the susceptibility to mesothelioma in Italian patients, pointing out the necessity to analyze a wider number of individuals, such as those used in the most recent genome wide studies, to deeper investigate the contribution of the inflammasome to the multifactorial mesothelioma.

## Additional files

Additional file 1: Figure S1. Histogram plot representing the distribution of *NLRP1* and *NLRP3* SNPs genotypes in subjects with documented asbestos exposure and death for pleural mesothelioma (AEM, dark bar) and individuals with documented asbestos exposure but death for other causes (AENM, grey bar). No statistically significant difference has been detected within AEM and AENM (see Table 1 in the main text of the article for p values). (DOCX 660 kb) Additional file 2: Table S1. Haplotype frequencies of the *NLRP1* and *NLRP3* polymorphisms in AEM and AENM individuals (DOCX 26 kb) Additional file 3: Figure S2. Haploview representation of the haploblocks found for *NLRP1* and *NRLP3* SNPs (DOCX 40 kb) Additional file 4: Table S2. Characteristics of selected malignant pleural mesothelioma (MPM) cases and controls in a Necropsy Series, Monfalcone Area 1980–2000. (DOCX 42 kb)

## Abbreviations

AB: Asbestos bodies
MPM: Malignant pleural mesothelioma
AEM: Subjects with documented asbestos exposure and death for pleural mesothelioma
AENM: Individuals with documented asbestos exposure but death for other causes
HWE: Hardy-Weinberg Equilibrium
