# Supplementary material for: NLRP1 and NLRP3 polymorphisms in mesothelioma patients and asbestos exposed individuals a population-based autopsy study from North East Italy
Source: Infect Agent Cancer. 2015 Aug 1;10:26. doi: 10.1186/s13027-015-0022-0 (PMC4521353; doi:10.1186/s13027-015-0022-0)
Supplement: Additional file 1: Figure S1. — Histogram plot representing the distribution of NLRP1 and NLRP3 SNPs genotypes in subjects with documented asbestos exposure and death for pleural mesothelioma (AEM, dark bar) and individuals with documented asbestos exposure but death for other causes (AENM, grey bar). No statistically significant difference has been detected within AEM and AENM (see Table 1 in the main text of the article for p values). (DOCX 660 kb) [file 13027_2015_22_MOESM1_ESM.docx]

**Additional file 1: Figure S1**

Histogram plot representing the distribution of *NLRP1* and *NLRP3* SNPs genotypes in subjects with documented asbestos exposure and death for pleural mesothelioma (AEM, dark bar) and individuals with documented asbestos exposure but death for other causes (AENM, grey bar).

No statistically significant difference has been detected within AEM and AENM (see Table 1 in the main text of the article for p values).
