# Supplementary material for: NLRP1 and NLRP3 polymorphisms in mesothelioma patients and asbestos exposed individuals a population-based autopsy study from North East Italy
Source: Infect Agent Cancer. 2015 Aug 1;10:26. doi: 10.1186/s13027-015-0022-0 (PMC4521353; doi:10.1186/s13027-015-0022-0)
Supplement: Additional file 2: Table S1. — Haplotype frequencies of the NLRP1 and NLRP3 polymorphisms in AEM and AENM individuals (DOCX 26 kb) [file 13027_2015_22_MOESM2_ESM.docx]

| **Additional file 2: Table S1**  Haplotype frequencies of the *NLRP1* and *NLRP3* polymorphisms in AEM and AENM individuals | | | | | | |
| --- | --- | --- | --- | --- | --- | --- |
| ***NLRP1* Haplo1** | | | | | | |
| rs9900356 | rs6502867 | rs9889625 | frequency | OR | 95% CI | p-value |
| T | T | A | 0.3893 | 1.00 |  |  |
| C | C | G | 0.1992 | 1.08 | 0.56 - 2.07 | 0.8185 |
| C | T | G | 0.0351 | 0.86 | 0.19 - 3.84 | 0.8427 |
| T | C | G | 0.0807 | 1.39 | 0.58 - 3.32 | 0.4648 |
| T | T | G | 0.2865 | 0.86 | 0.49 - 1.49 | 0.5890 |
| rare haplotypes | | | 0.0091 | 0.85 | 0.05 - 14.57 | 0.9107 |
| ***NLRP1* Haplo2** | | | | | | |
| rs12150220 | rs2670660 |  | frequency | OR | 95% CI | p-value |
| A | A |  | 0.4592 | 1.00 |  |  |
| A | G |  | 0.0486 | 1.28 | 0.37 - 4.38 | 0.6994 |
| T | A |  | 0.0408 | 1.34 | 0.35 - 5.04 | 0.6687 |
| T | G |  | 0.4514 | 1.05 | 0.63 - 1.73 | 0.8633 |
| ***NLRP3*** | | | | | | |
| rs35829419 | rs10754558 |  | frequency | OR | 95% CI | p-value |
| C | C |  | 0.5859 | 1.00 |  |  |
| A | G |  | 0.0781 | 1.26 | 0.55-2.88 | 0.5860 |
| C | G |  | 0.3359 | 1.08 | 0.64-1.82 | 0.7689 |
