# Supplementary material for: NLRP1 and NLRP3 polymorphisms in mesothelioma patients and asbestos exposed individuals a population-based autopsy study from North East Italy
Source: Infect Agent Cancer. 2015 Aug 1;10:26. doi: 10.1186/s13027-015-0022-0 (PMC4521353; doi:10.1186/s13027-015-0022-0)

**Additional file 3: Figure S2**

Haploview representation of the haploblocks found for *NLRP1* and *NRLP3* SNPs


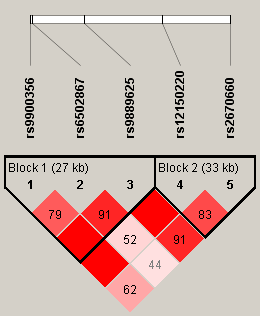

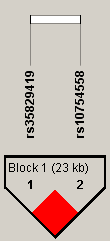

Supplement: Additional file 3: Figure S2. — Haploview representation of the haploblocks found for NLRP1 and NRLP3 SNPs (DOCX 40 kb) [file 13027_2015_22_MOESM3_ESM.docx]
