# Supplementary material for: NLRP1 and NLRP3 polymorphisms in mesothelioma patients and asbestos exposed individuals a population-based autopsy study from North East Italy
Source: Infect Agent Cancer. 2015 Aug 1;10:26. doi: 10.1186/s13027-015-0022-0 (PMC4521353; doi:10.1186/s13027-015-0022-0)
Supplement: Additional file 4: Table S2. — Characteristics of selected malignant pleural mesothelioma (MPM) cases and controls in a Necropsy Series, Monfalcone Area 1980–2000. (DOCX 42 kb) [file 13027_2015_22_MOESM4_ESM.docx]

**Additional file 4: Table S2**

Characteristics of selected malignant pleural mesothelioma (MPM) cases and controls in a Necropsy Series, Monfalcone Area 1980–2000.

|  | **Study group (AEM)** | **Controls ( AENM)** |
| --- | --- | --- |
| **Gender** |  |  |
| Men | 61 | 57 |
| Women | 8 | 2 |
| **Age at death** |  |  |
| years (mean+ Std. Error) | Mean 70,2+1,3 | 80,1+0,5 |
| **Cause of death** |  |  |
| MPM | 69 | - |
| Non neoplastic  (not asbestos related: pneumonia, pulmonary embolism, edema, stroke and myocardial infarction) | - | 32 |
| Neoplastic  (not asbestos related: prostate, liver, mouth, large bowel, stomach, skin, bladder and adrenals) | - | 27 |
| **Pleural plaques** |  |  |
| Absent | 6 | 0 |
| Class1 | 13 | 0 |
| Class2 | 21 | 27 |
| Class3 | 26 | 32 |
| **Lung AB counts**  **(n°/g dry lung tissue)** |  |  |
| 0-999 | 7 | 0 |
| 1.000-9.999 | 27 | 0 |
| 10.000-99.999 | 21 | 31 |
| 100.000-1.000.000 | 10 | 12 |
| Not available | 4 | 16 |
| **Occupational exposure data** |  |  |
| Shipbuilding industry | 39 | 28 |
| Other industries (textile) | 5 | 1 |
| Navy and merchant marine | 4 | 0 |
| Domestic exposure | 4 | 0 |
| Not available | 17 | 30 |
| **Total** | **69** | **59** |

**Study population: subjects exposed to asbestos who died for MPM (Asbestos Exposed MPM=AEM)**

As study population we selected 69 subjects died from MPM (asbestosis free) with objective signs confirming asbestos exposure: pleural plaques and/or presence of AB in routine lung sections and (when available) a documented occupational history of asbestos exposure.

**Control population: individuals exposed to asbestos who died for other causes (Asbestos Exposed Non MPM=AENM)**

Exposed subjects with the highest MPM-free follow-up were selected as controls in this study. We selected 59 subjects with objective signs confirming asbestos exposure (see above), who didn’t develop pleural or peritoneal malignant mesothelioma, lung carcinoma and lung asbestosis, and died after age 75 (≥75 years) of other causes.
